# Anatomy, Descriptive and Surgical

**Published:** 1859-03

**Authors:** 


ART. VI.—*Anatomy, Descriptive and Surgical.* By HENRY GRAY, F.R.S., Lecturer on Anatomy at St. George's Hospital. Super-royal 8vo., pp. 750. John W. Parker & Son: London. 1858.

FOR this truly admirable work the profession is indebted to the distinguished author of Gray on the Spleen, and Dr. H. V. Carter, Designer and editor *a latere.*

The vacancy it fills has been long felt to exist in this country. Anatomical works of all kinds abound, it is true; but it is no dispraise to say that as yet the most comprehensive among them are to be recognized as the works of men who have been laborers in their own special departments of the science. Quain, for instance, as edited by Sharpey and Ellis, is thorough as far as it goes, and gives us an admirably-systematized arrangement of the "Elements of Anatomy." We are also indebted to various other authors for their excellent operative surgeries. Mr. Gray, however, writes throughout with both branches of his subject in view. His description of each particular part is followed by a notice of its relations to the parts with which it is connected, and this, too, sufficiently ample for all the purposes of the operative surgeon. After describing the bones and muscles, he gives a concise statement of the fractures to which the bones of the extremities are most liable, together with the amount and direction of the displacement to which the fragments are subjected by muscular action. The section on arteries is remarkably full and accurate. Not only is the surgical anatomy given of every important vessel, with directions for its ligation, but at the end of the description of each arterial trunk we have a useful summary of the irregularities which may occur in its origin, course, and termination.

In that philosophic serial, the Transactions of the Pickwick Club, Mr. C. Dickens notes the case of an author, who, having occasion to prepare an article on Chinese metaphysics, consulted the British Encyclopedia. He read for China under the letter C, and metaphysics under the letter M, "and combined his information, sir." We shall not consume a page to answer those who are of course prepared to ask, Why not keep "each department of our study" separate? Why not have two sets of text-books—two sets of plates—two sets of thoughts, if you please, at all times for the student—to be purchased by him at separate seasons, each separate item in its order? Why should not the student himself collect the facts in separate parcels, as they were collected by his teachers before him? They did not mix up the two when they were at work with their sketch-books and note-books and their subject on the table, nor did they accumulate their memoranda of demonstrative anatomy upon the occasions when they were performing operations in the amphitheatre in the presence only of their suffering patient and an admiring audience. Then why should he? Why, indeed? Nevertheless, such has been our experience of the proper course of teaching. Arrest the attention of your class, say we, as quickly and effectually as you can; let them get into the heart of your subject in any way they prefer, and which proves easiest and most direct to them. There is no essential reason whatever why their difficulties should repeat your difficulties.

We have a capital illustration of this furnished in the plates of the volume before us. Mr. Holden, in his recent work on "Human Osteology," having marked the muscular attachments on the bone in dotted lines, and having written the name of the muscle on the surface of attachment, instead of below with a numeral reference, as heretofore practiced, Dr. Carter has carried out this idea and applied it to all other portions of the body, so that every muscle, artery, and nerve has its name written upon it. By written, we mean printed—clearly, distinctly, legibly. It is true, this mars somewhat the artistic effect of the plates, but it renders them infinitely more useful and easy of reference, the student being able to turn directly from the plate to the subject, instead of being bewildered by having to hunt up marginal notes, as formerly. The muscle or bone labeled this or that, he remembers in connection with other bones and muscles also labeled in like manner. He sees them, and thinks of them by their proper names. His associations and random recollections are with useful words, and not with figures.

We cannot too highly praise these illustrations. Those of Holden should not be compared with them. Holden's are lithographic, the dotted lines representing the muscular attachments are in colors, and no effort has been spared to give them artistic finish and expression. But Carter's are wood-cuts—lie in the body of the letter-press, are cheaper, of course, and more readily accessible. They often partake of the character of diagrams, rather than finished drawings; as in the case of the plates of the brain and nervous system, to which we would particularly refer. But they are uniformly clear and accurate, and always succeed in telling their story. They are the best wood-cut anatomical illustrations we have seen.

They are original, too, as the preface tells us, either copied from or corrected by recent dissections, made jointly by the author and Dr. Carter. Our eyes are not wearied by looking over for the hundredth time the retouched plates of Wilson, nor shocked to discover a piracy in the first degree from some continental work. With the exception of those relating to the veins of the spine, for which full credit is given to Breschet, and those illustrating the lymphatic system, which the author himself states to be taken from Mascagni, the cuts of this book are not copies of those of any other anatomy.

A word as to the letter-press. Mr. Gray has done his part well. His style is in general good, is only occasionally obscure, and more French and Scotch than English. The difficulty of correcting the proofs of such a book is very great, and we shall be prepared to see a few inaccuracies, such as, *is* for *it*, *is* for *are*, and *are* for *is*, oblique line of *tibia* for oblique line of *fibula*, "the middle bifurcation of the linea aspera," corrected in subsequent editions. As a Briton, too, Mr. Gray is to be thanked for having heard of such a man as Cruveilhier. In his description of the muscles he has adhered to the English method of classification, and describes with national accuracy the interminable layers of muscles on the back; but in speaking of the muscles of the anterior part of the thigh, he does mention the impossibility of separating the crurœus from the vastus internus, and the analogy which exists between the mass usually described as quadriceps femoris and the triceps extensor brachialis. It is noble thus candidly to admit that there is a continental city named Paris, the seat of a medical school, where no other classifications are in use than those of M. Cruveilhier.

What a labor to get up such a work in such a style! The type is good, paper excellent, price rather cheap than otherwise. We trust sincerely that Gray and Carter's Anatomy will not be republished in this country without a license from the author.

